# Lung-Protective Ventilation Strategies for Relief from Ventilator-Associated Lung Injury in Patients Undergoing Craniotomy: A Bicenter Randomized, Parallel, and Controlled Trial

**DOI:** 10.1155/2017/6501248

**Published:** 2017-07-05

**Authors:** Chaoliang Tang, Juan Li, Shaoqing Lei, Bo Zhao, Zhetao Zhang, Wenting Huang, Si Shi, Xiaoqing Chai, Chaoshi Niu, Zhongyuan Xia

**Affiliations:** ^1^Department of Anesthesiology, Renmin Hospital of Wuhan University, Wuhan, China; ^2^Department of Anesthesiology, Anhui Provincial Hospital of Anhui Medical University, Hefei, China; ^3^Department of Pharmacy, Anhui Provincial Hospital of Anhui Medical University, Hefei, China; ^4^Department of Neurosurgery, Anhui Provincial Hospital of Anhui Medical University, Hefei, China

## Abstract

Current evidence indicates that conventional mechanical ventilation often leads to lung inflammatory response and oxidative stress, while lung-protective ventilation (LPV) minimizes the risk of ventilator-associated lung injury (VALI). This study evaluated the effects of LPV on relief of pulmonary injury, inflammatory response, and oxidative stress among patients undergoing craniotomy. Sixty patients undergoing craniotomy received either conventional mechanical (12 mL/kg tidal volume [V_T_] and 0 cm H_2_O positive end-expiratory pressure [PEEP]; CV group) or protective lung (6 mL/kg V_T_ and 10 cm H_2_O PEEP; PV group) ventilation. Hemodynamic variables, lung function indexes, and inflammatory and oxidative stress markers were assessed. The PV group exhibited greater dynamic lung compliance and lower respiratory index than the CV group during surgery (*P* < 0.05). The PV group exhibited higher plasma interleukin- (IL-) 10 levels and lower plasma malondialdehyde and nitric oxide and bronchoalveolar lavage fluid, IL-6, IL-8, tumor necrosis factor-*α*, IL-10, malondialdehyde, nitric oxide, and superoxide dismutase levels (*P* < 0.05) than the CV group. There were no significant differences in hemodynamic variables, blood loss, liquid input, urine output, or duration of mechanical ventilation between the two groups (*P* > 0.05). Patients receiving LPV during craniotomy exhibited low perioperative inflammatory response, oxidative stress, and VALI.

## 1. Introduction

Mechanical ventilation (MV) is the most effective means of providing respiratory support in the operating room and intensive care unit (ICU). Annually, approximately 2.5 million patients rely on MV during surgery [1]. Pulmonary complications—including atelectasis, acute lung injury, pneumonia, and infection—associated with MV are major contributors to increased patient morbidity and mortality [2, 3]. According to current evidence, potential harmful effects of conventional MV with ventilator parameters such as tidal volume (V_T_) > 6 mL/kg predicted body weight (PBW) and exposure to high airway pressure even during short-term treatment have been shown to be correlated with systemic inflammation and development of ventilator-associated lung injury (VALI) because of cyclic alveolar atelectasis and strain. High V_T_ ventilation helps maintain the partial pressure of oxygen in arterial blood (PaO_2_) at normal levels; however, it can cause excessive expansion of the lungs with normal oxygenation. General anesthesia with anesthetics and neuromuscular blocking agents can cause changes in pulmonary surfactants and diaphragmatic position, and inappropriate ventilator settings are likely to cause air pressure injury and atelectasis, leading to postoperative inflammation [4, 5]. Complications in MV due to surgical trauma can also lead to postoperative local, and even systemic, inflammatory responses and pulmonary complications [6, 7]. In addition, there is evidence that MV of healthy lungs can induce upregulation of cytokines, leading to proinflammatory cytokine gene transcription, predisposing the organism to infection, and oxidative stress [8].

Lung-protective ventilation (LPV) strategies have recently been developed to reduce ventilator-associated lung tissue injury and simultaneously improve systemic oxygenation [9, 10]. Lung-protective ventilation regulates positive end-expiratory pressure (PEEP), maintains a greater number of pulmonary alveoli in an open state, avoids elevation of end-expiratory lung volume, helps maintain target V_T_, and alleviates injuries caused by elevated lung volume and abnormal V_T_. Positive end-expiratory pressure can prevent the collapse of open pulmonary alveoli, maintain lung volume and function of pulmonary surfactants, and reduce the shear stress caused by repeated/loss of alveolar or bronchiolar recruitment(s) [11]. In comparison with ventilation with higher V_T_ (10–12 mL/kg PBW) without PEEP, intraoperative LPV with lower V_T_ (6–8 mL/kg PBW) with PEEP has been shown to decrease postoperative mortality [12–14].

Relative to other forms of surgery, neurosurgery takes a longer time and requires hyperventilation for the patient. However, regular long-term clinical ventilation with high V_T_ could not only decrease the cardiac output and blood pressure but also cause serious VALI [15, 16]. In addition, in neurosurgery, since the surgical site is far away from the chest, the surgical procedure has a relatively low direct influence on breathing, and the circulation is relatively stable during perioperative ventilation. Although LPV is widely used in clinical settings, particularly in one-lung ventilation and abdominal surgery, there have been no studies on LPV in patients without lung pathology undergoing craniocerebral surgery. We, therefore, conducted this prospective, randomized, double-blind study in two tertiary-care hospitals in Hefei and Wuhan, China, to explore the effect of LPV with low V_T_ and PEEP on intraoperative pulmonary injury, inflammation, and oxidative stress in patients undergoing craniotomy.

## 2. Materials and Methods

### 2.1. Subjects

This prospective, randomized, double-blind clinical trial was approved by the local Clinical Research Ethics Committees (2014 [59]) and was registered in the Chinese Clinical Trial Registry (ChiCTR; registration number ChiCTR-IPR-16008029). Written informed consent was obtained from all patients. Patients of either sex with American Society Anesthesiologists' physical status I-II, age between 18 and 70 years, and normal preoperative pulmonary function who were scheduled for craniocerebral surgery were recruited.

The exclusion criteria were as follows: presence of bronchial infection, obstructive or restrictive lung disease, asthma and sleep apnea syndrome, severe hypertension and cardiovascular diseases, liver or kidney dysfunction, history of second- or third-degree heart block and ischemic heart diseases, and body mass index >35 kg/m^2^.

Patients were assigned to one of two study groups—the CV (conventional MV with 12 mL/kg V_T_ and 0 cm H_2_O PEEP) or PV (protective lung ventilation with 6 mL/kg V_T_ and 10 cm H_2_O PEEP) group (*n* = 30, each)—using a random number table, which was prepared by a statistician who was unaware of the purpose of the study.

### 2.2. Surgical Procedure

Patients were brought to the surgical room without premedication. Standard monitoring procedure involved five-lead electrocardiography, monitoring of oxygen saturation, and noninvasive blood pressure evaluation. The anesthetist prepared a 50 mL syringe containing 4 *μ*g/mL of dexmedetomidine. A 20-gauge intravenous cannula was inserted into the dorsum of the left hand of the patients. All patients were administered 8–10 mL/kg hydroxyethyl starch 130/0.4 (Voluven). Once bispectral index (BIS) monitoring was commenced, the patients were administered 0.6 *μ*g/kg dexmedetomidine; the dosage was then changed to allow continuous infusion of 0.3 *μ*g/kg/h dexmedetomidine for maintenance after 15 min.

Before induction of anesthesia, preoxygenation was ensured by delivery of 100% oxygen through a facial mask for no less than 3 min. Following insertion of an arterial line under local anesthesia, general anesthesia was induced with 0.3 mg/kg etomidate, 0.5 *μ*g/kg sufentanil, and 1.2 mg/kg rocuronium. Manual facemask ventilation was continued for no less than 3 min until the jaw was relaxed; the BIS was maintained at <50 to allow tracheal intubation. Anesthesia was maintained using the Datex Ohmeda S/5 Avance Anesthesia Machine (S/5; Datex Ohmeda, Helsinki, Finland). Mechanical ventilation was commenced with 60% fraction of inspired O_2_ (FiO_2_) and 6–8 mL/kg V_T_ at a frequency of 10–12 times/min to maintain partial pressure of CO_2_ (EtCO_2_) within the normal range. Sevoflurane (1%) was administered as an inhalant, and a target-controlled anesthesia system (TCI) (Alaris MK III, CareFusion, Rolle, Switzerland) was used for administering remifentanil and propofol in order to maintain the BIS between 40 and 60 and to ensure that variations in mean arterial pressure and heart rate (HR) did not exceed 20% of the baseline values. Next, a central venous catheter (jugular vein) and an indwelling bladder catheter were inserted. Following stabilization of hemodynamic parameters after intubation, the ventilation strategies were changed according to the group allocation—patients in the CV group were ventilated with 12 mL/kg V_T_ and 0 cm H_2_O PEEP, while those in the PV group were ventilated with 6 mL/kg V_T_ and 10 cm H_2_O PEEP.

Hypotension (>20% decrease in baseline blood pressure) was treated with 5 mg intravenous ephedrine or 40 *μ*g phenylephrine, while bradycardia was treated with 0.5 mg intravenous atropine. Standardized anesthesiological management was practiced.

Following skull flap fixation, the patients were administered 1 mg/kg of tramadol and 10 mg of azasetron, and administration of sevoflurane and dexmedetomidine was stopped. After incision closure and withdrawal of bronchoalveolar lavage fluid (BALF), TCI administration of anesthetics was stopped. In both study groups, before the patients resumed spontaneous breathing and responded to simple commands, ventilation was switched to the synchronized intermittent MV (SIMV) mode with 0 cm H_2_O PEEP to provide assistance. Reversal of neuromuscular blockade was achieved with 50 *μ*g/kg neostigmine and 20 *μ*g/kg atropine. After ensuring compliance with the standard extubation criteria [17], the endotracheal tube was removed.

After extubation, patients were transferred to the post anesthesia care unit (PACU) and monitored for a minimum of 1 h postoperatively. Afterwards, all patients were transferred to the Neurosurgery ICU for further monitoring and routine treatment over the next 24 h.

### 2.3. Outcome Measures

Systolic blood pressure (SBP) and diastolic blood pressure (DBP) and HR were recorded at five time points: T1—just before changing the ventilation strategy following stabilization of hemodynamic parameters after intubation; T2 and T3—1 and 3 h, respectively, after changing the ventilation strategy; T4—end of surgery; and T5—immediately after extubation. Intraoperative blood loss, liquid input, urine output, and durations of MV and surgery were also recorded.

After induction of general anesthesia, peak (Ppeak) and plateau (Pplat) inspiratory pressure as well as PEEP were monitored continuously with the Datex-Ohmeda S/5 Avance Anesthesia Machine. Dynamic lung compliance (Cldyn) of the respiratory system was calculated using the following standard formula: Cldyn = VT/(Ppeak − PEEP) [18]. For calculating the oxygen (OI) and respiratory (RI) indexes, 2 mL of arterial blood was withdrawn from each patient at T1, T2, T3, T4, and T5. The OI and RI were assessed using an automatic blood gas analyzer, in accordance with the following formulas: OI = PaO_2_/FiO_2_; RI = P(A − a)O_2_/PaO_2_ = {[(PB − PH_2_O × FiO_2_ − PaCO_2_) − PaO_2_]/PaO_2_. Here, P(A–a) O_2_ indicated the alveolar-arterial gradient; PB, atmospheric pressure; PH_2_O, saturated vapor pressure; FiO_2_, inhaled oxygen concentration (%); and PaCO_2_, arterial carbon dioxide partial pressure [19].

The primary outcome measures were differences in inflammatory and oxidative stress markers in the plasma and BALF. To this end, 3 mL venous blood and BALF were withdrawn immediately before changing the ventilation strategy after intubation (precontinuous ventilatory support [pre-CVS]) and immediately before switching the ventilation strategy to the SIMV mode with 0 cm H_2_O PEEP (pre-SIMV) in both groups. Bronchoalveolar lavage fluid was withdrawn using a previously described method [20]. Plasma and BALF levels of interleukin- (IL-) 6, IL-8, tumor necrosis factor-alpha (TNF-*α*), IL-10, malondialdehyde (MDA), nitric oxide (NO; NO_2_^−^/NO_3_^−^), and superoxide dismutase (SOD) were assessed by enzyme-linked immunosorbent assay.

### 2.4. Statistical Analysis

Power calculation was performed on the basis of respiratory index. A pilot study involving 6 patients at our center found the mean ± standard deviation (SD) of respiratory index to be 0.95 ± 0.18. A sample size of 50 patients was required to observe a clinically significant reduction of 20% in respiratory index at a power of 95% and two-sided significance level of 0.05. To compensate for the possibility of dropout, we recruited 60 patients (30 patients per group).

All measurement indexes were expressed as mean ± SD/standard error of the mean or number (%). After analysis of normality of data distribution, normally distributed data were compared by the independent sample *t*-test. Unpaired quantitative variables were evaluated by the Student *t*-test and analysis of variance. The Mann–Whitney *U* test was employed for intergroup comparison, and the Wilcoxon signed-rank test for comparison between different time points within the same group. Intergroup comparison of categorical variables was performed by the chi-square test. Values of *P* < 0.05 were considered statistically significant. All data were statistically analyzed by statisticians using the SPSS 13.0 software package (IBM Corp., Armonk, NY, USA) in line with the intention-to-treat principle.

## 3. Results

**Figure 1:**
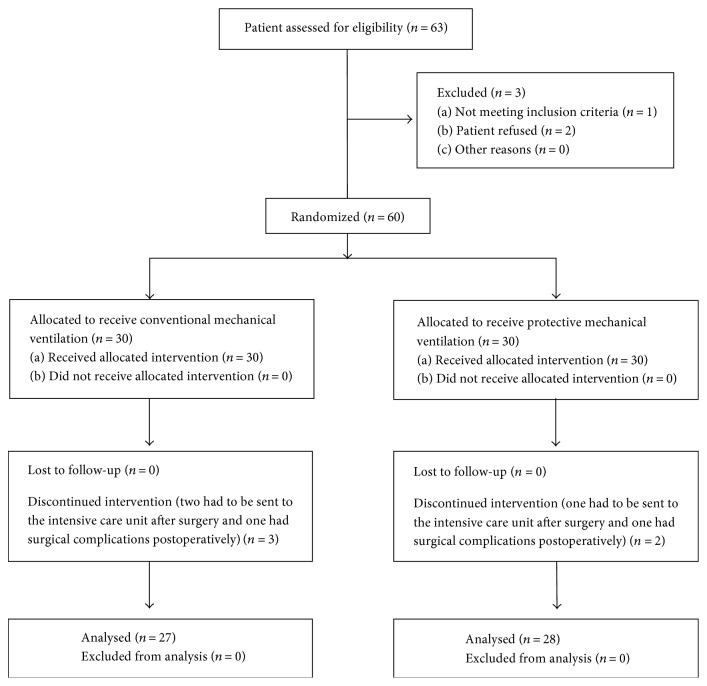
Flow diagram of patient recruitment.

Between March and December 2016, 63 patients were recruited to this study. Three patients in the CV group dropped out from the investigation—while two patients had to be transferred to the ICU after surgery, one experienced postoperative complications. In the PV group, two patients dropped out from the investigation—while one patient had to be transferred to the ICU after surgery, the other experienced postoperative complications. Finally, 55 patients completed the study—CV group, 27; PV group, 28 (Figure 1). There were no significant differences in demographic data, surgical characteristics, or intraoperative variables between the two groups (Tables 1 and 2).

**Figure 2:**
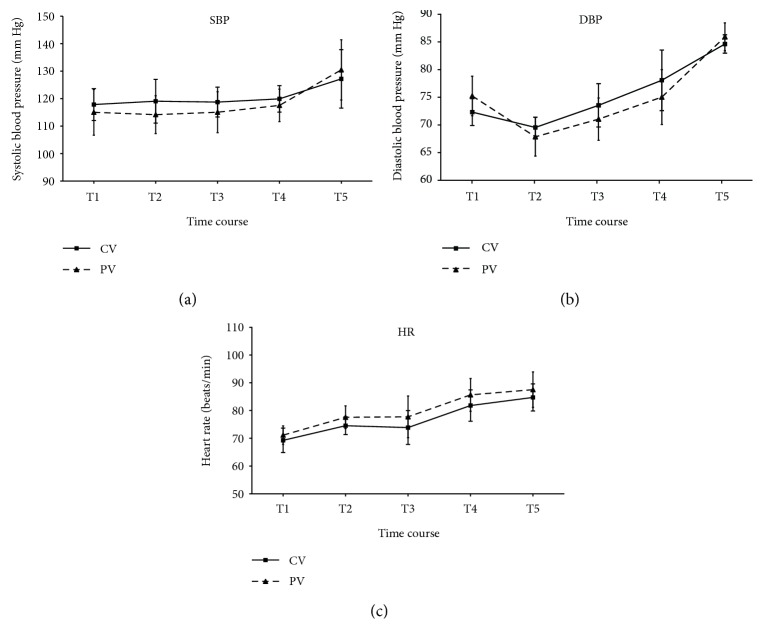
Changes in hemodynamic variables among patients who were administered conventional mechanical ventilation with 12 mL/kg tidal volume (V_T_) and 0 cm H_2_O positive end-expiratory pressure (PEEP) (CV group) or protective lung ventilation with 6 mL/kg V_T_ and 10 cm H_2_O PEEP (PV group) during surgery. Bars indicate the standard deviation. The time points for measurements were T1—just before changing the ventilation strategy following stabilization of hemodynamic parameters after intubation; T2 and T3—1 and 3 h, respectively, after changing the ventilation strategy; T4—end of surgery; and T5—immediately after extubation.

There were also no significant differences in baseline SBP, DBP, or HR between the two groups. Of note, during the observation period before the end of surgery, patients in the PV group exhibited greater reductions in SBP and DBP and a greater increase in HR than patients in the CV group. However, the differences were not significant (*P* > 0.05; Figure 2).

**Figure 3:**
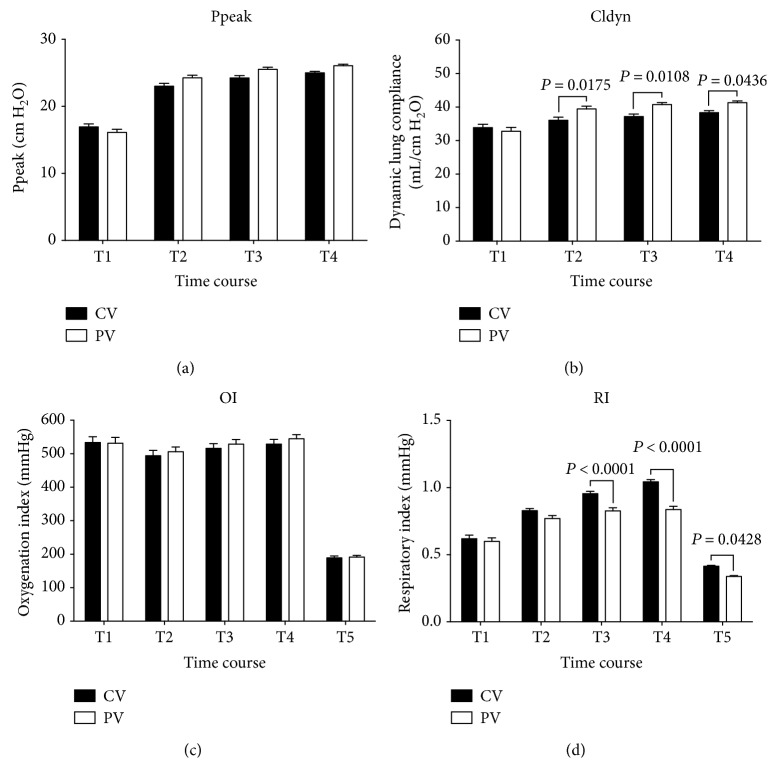
Changes in Ppeak, Cldyn, OI, and RI levels among patients receiving conventional mechanical or protective lung ventilation during surgery. Values are given as mean ± standard error of the mean. Ppeak, peak inspiratory pressure; Cldyn, dynamic lung compliance; OI, oxygen index; RI, respiratory index; CV group, conventional mechanical ventilation with 12 mL/kg tidal volume (V_T_) and 0 cm H_2_O positive end-expiratory pressure (PEEP); PV group, protective lung ventilation with 6 mL/kg V_T_ and 10 cm H_2_O PEEP.

The OI and Ppeak were comparable and within normal limits in both groups. Although the PV group tended to exhibit higher OI values from intubation to extubation than the CV group, the differences did not reach statistical significance (*P* > 0.05). In the PV group, the Cldyn levels at T2, T3, and T4 were 39.44 ± 0.84, 40.75 ± 0.57, and 41.31 ± 0.51 mL/cm H_2_O, respectively, which differed significantly from the corresponding values in the CV group—36.06 ± 0.93, 37.19 ± 0.73, and 38.31 ± 0.62 mL/cm H_2_O, respectively (*P* = 0.0175, 0.0108, and 0.0436, resp.; Figure 3(b)). In the PV group, the RI values at T3, T4, and T5 were 0.83 ± 0.093, 0.84 ± 0.097, and 0.34 ± 0.026 mmHg, respectively, which differed significantly from the corresponding values in the CV group—0.96 ± 0.069, 1.04 ± 0.065, and 0.41 ± 0.031 mmHg (*P* < 0.0001, *P* < 0.0001, and *P* = 0.0428, resp.; Figure 3(d)).

**Figure 4:**
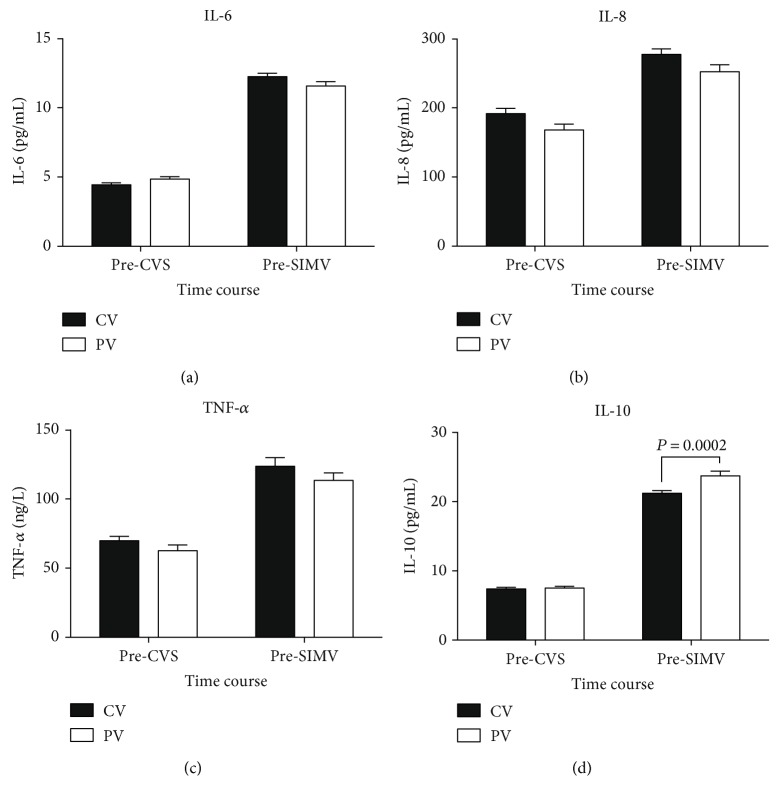
Changes in plasma interleukin- (IL-) 6, IL-8, tumor necrosis factor-alpha (TNF-*α*), and IL-10 levels among patients receiving conventional mechanical or protective lung ventilation during surgery. Values are expressed as mean ± standard error of the mean. CVS, continuous ventilatory support; SIMV, synchronized intermittent mechanical ventilation; CV group, conventional mechanical ventilation with 12 mL/kg tidal volume (V_T_) and 0 cm H_2_O positive end-expiratory pressure (PEEP); PV group, protective lung ventilation with 6 mL/kg V_T_ and 10 cm H_2_O PEEP.

There were no significant differences in pre-CVS or pre-SIMV plasma concentrations of IL-6, IL-8, and TNF-*α* different between the two groups; however, in both groups, the pre-SIMV concentrations were significantly higher than the pre-CVS concentrations. The pre-SIMV concentrations of IL-10 in both groups were also significantly higher than the pre-CVS IL-10 concentrations; however, the pre-SIMV IL-10 concentration in the PV group was significantly higher than that in the CV group (*P* = 0.0002; Figure 4(d)).

**Figure 5:**
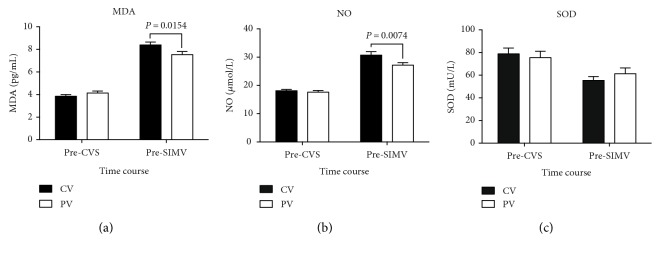
Changes in plasma malondialdehyde (MDA), nitric oxide (NO), and superoxide dismutase (SOD) levels among patients receiving conventional mechanical or protective lung ventilation during surgery. Values are given as means ± standard error of the mean. CVS, continuous ventilatory support; SIMV, synchronized intermittent mechanical ventilation; CV group, conventional mechanical ventilation with 12 mL/kg tidal volume (V_T_) and 0 cm H_2_O positive end-expiratory pressure (PEEP); PV group, protective lung ventilation with 6 mL/kg V_T_ and 10 cm H_2_O PEEP.

Both groups exhibited significant increases in plasma concentrations of MDA and NO over time; however, the PV group exhibited significantly lower pre-SIMV MDA and NO concentrations than the CV group (*P* = 0.0154 and 0.0074, resp.; Figures 5(a) and 5(b), resp.). In the CV group, the pre-SIMV SOD concentration (55.31 ± 3.47 mU/L) was significantly lower than the pre-CVS SOD concentration (78.87 ± 5.04 mU/L; *P* = 0.0056). However, there were no significant differences in SOD levels between the two groups (*P* = 0.6279; Figure 5(c)).

**Figure 6:**
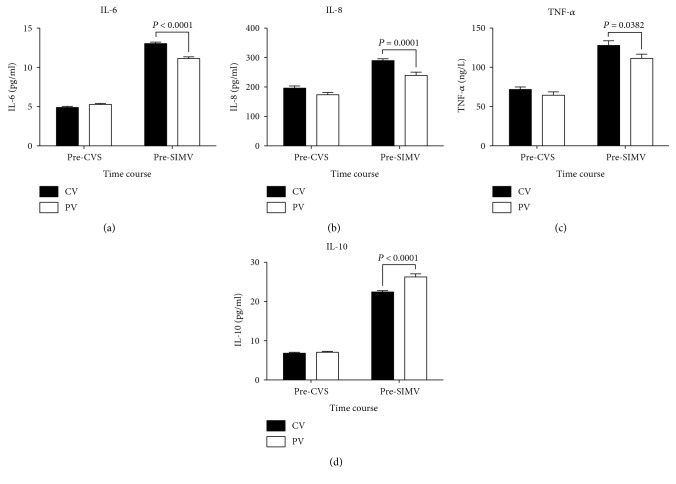
Changes in interleukin- (IL-) 6, IL-8, tumor necrosis factor-alpha (TNF-*α*), and IL-10 levels in bronchoalveolar lavage fluid among patients receiving conventional mechanical or protective lung ventilation during surgery. Values are expressed as mean ± standard error of the mean. CVS, continuous ventilatory support; SIMV, synchronized intermittent mechanical ventilation; CV group, conventional mechanical ventilation with 12 mL/kg tidal volume (V_T_) and 0 cm H_2_O positive end-expiratory pressure (PEEP); PV group, protective lung ventilation with 6 mL/kg V_T_ and 10 cm H_2_O PEEP.

In both groups, the pre-SIMV BALF concentrations of IL-6, IL-8, TNF-*α*, and IL-10 were all significantly higher than the corresponding pre-CVS concentrations. The pre-SIMV concentrations of IL-6, IL-8, and TNF-*α* in the PV group were significantly lower than those in the CV group (*P* < 0.0001, *P* = 0.0001, and *P* = 0.0382, resp.; Figures 6(a), 6(b), and 6(c), resp.). In contrast, the pre-SIMV concentration of IL-10 in the PV group was significantly higher than that in the CV group (*P* < 0.0001; Figure 6(d)).

**Figure 7:**
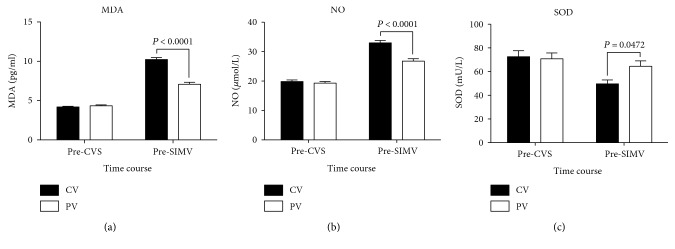
Changes in malondialdehyde (MDA), nitric oxide (NO), and superoxide dismutase (SOD) levels in bronchoalveolar lavage fluid among patients receiving conventional mechanical or protective lung ventilation during surgery. Values are given as mean ± standard error of the mean. CVS, continuous ventilatory support; SIMV, synchronized intermittent mechanical ventilation; CV group, conventional mechanical ventilation with 12 mL/kg tidal volume (V_T_) and 0 cm H_2_O positive end-expiratory pressure (PEEP); PV group, protective lung ventilation with 6 mL/kg V_T_ and 10 cm H_2_O PEEP.

Both groups exhibited significant increases in BALF concentrations of MDA and NO over time; however, the PV group exhibited significantly lower pre-SIMV MDA and NO concentrations than the CV group (*P* < 0.0001, both; Figures 7(a) and 7(b), resp.). In the CV group, the pre-SIMV concentration of SOD (49.69 ± 3.31 mU/L) was significantly lower than the pre-CVS concentration (72.61 ± 5.06 mU/L; *P* = 0.0033). The pre-SIMV SOD concentration in the PV group was higher than that in the CV group (*P* = 0.0472; Figure 7(c)).

## 4. Discussion

This randomized, double-blind, comparative study was undertaken to evaluate the effects of LPV during craniotomy on pulmonary injury, inflammatory response, and oxidative stress. Our principal findings suggest that LPV during craniotomy effectively reduces intraoperative pulmonary injury—as evident from the relatively low inflammatory response and oxidative stress—without inducing clinically relevant hemodynamic changes.

Respiratory index is an important monitoring index of lung diffusion function. It can also accurately reflect the degree of pulmonary injury—the higher the RI, the more serious the pulmonary injury. Dynamic lung compliance reflects the degree of lung compliance; it could be affected by pulmonary surfactant activity, atelectasis, bronchospasm, and pulmonary edema [19, 21]. In case of OI, lower values are better, as can be inferred from the equation. Improvement in oxygenation allows patients to achieve higher PaO_2_ at a lower fraction of inspired oxygen. In our study, the Ppeak and OI values were comparable and within normal limits in both groups, and all patients received sufficient oxygen supply. The PV group exhibited higher Cldyn levels and lower RI than the CV group; although the corresponding differences were statistically significant, their clinical significance remains to be further evaluated. Overall, patients without lung injury requiring MV will benefit from LPV.

Tidal volume limits of 6 mL/kg or less PBW might ensure less mechanical stress on the alveolar-capillary membrane, thus preventing alveolar overdistension and improving alveolar stability. Furthermore, appropriate PEEP levels can also help stabilize the alveoli and avoid derecruitment by increasing the end-expiratory lung volume. A previous study has indicated that LPV might be beneficial for the lungs; it may impair the cardiovascular system for the use of PEEP. This effect may be mainly be brought about by the reduction of venous return and cardiac output and use of fluids and vasopressors [12].

In the present study, relative to patients in the CV group, those in the PV group exhibited greater reductions in SBP and DBP and a greater increase in HR during the observation period before the end of surgery. However, these parameters were all within normal limits, and the differences were statistically insignificant. This trend was maintained in terms of intraoperative liquid input and urine output as well. In addition, the risks and benefits of LPV should be balanced in each patient.

Sustained mechanical distension of the lungs due to hyperinflation, contributes to volutrauma, barotrauma, and biotrauma, which may cause intensive stress leading to direct cell injury. The resulting consequences are capillary damage and pulmonary edema [22], localized tissue inflammation, sustained increase in local and systemic release of lung borne inflammatory markers, and procoagulant changes in alveolar fluid [23]. Proinflammatory cytokines (e.g., IL-6, IL-8, and TNF-*α*) and anti-inflammatory cytokines (e.g., IL-10) are important mediators of inflammation; they play an essential role in lung inflammation models [24–26]. During inflammation, TNF-*α* is released, which subsequently enhances mononuclear cell and macrophage activities and increases cell adhesive factor expression, thereby inducing tissue injury. Interleukin-8 induces accumulation of white blood cells in inflammatory tissues. In contrast, IL-10 inhibits transcription of proinflammatory cytokines (TNF-*α*, IL-6, and IL-8) and reduces white blood cell adhesion to vascular endothelial cells in the lungs, resulting in the attenuation of lung injury [19].

Clinical evidence now demonstrates that delivery of high V_T_ (>10 mL/kg PBW) through MV is associated with localized lung inflammation in patients without preexisting lung injury [27–29]. Lung-protective ventilation could prevent both localized and generalized lung inflammation, thereby attenuating intraoperative pulmonary injuries. In the present study, patients of both groups exhibited significant increases in plasma and BALF IL-6, IL-8, TNF-*α*, and IL-10 concentrations at T4. Relative to the CV group, the PV group exhibited lower proinflammatory cytokine levels and higher anti-inflammatory cytokine levels in BALF at T4. In contrast, while the PV group exhibited a significantly higher plasma IL-10 concentration than the CV group, there were no significant differences in plasma IL-6, IL-8, and TNF-*α* concentrations between the two groups.

As already known, conventional MV for a short duration of 5 h promotes localized bronchoalveolar inflammatory changes in patients without preexisting lung injury [29]. In addition, the biotrauma invoked by pulmonary proinflammatory markers can also induce a systemic inflammatory response [30, 31]. In the present study, the mean duration of MV was more than 6 h, by which time, only localized bronchoalveolar inflammation had been significantly promoted. One possible reason is that the duration of MV was not long enough to promote generalized inflammation [32]; another reason might be that the use of anesthetics such as dexmedetomidine—which was initiated soon after the transfer of patients to the operation room—prevented a stress response during the initial hours of MV [17]. Since the deleterious effects of MV are, in part, dependent on the duration of MV, LPV should be initiated early during the ventilation process.

The lungs are among the key organs for development of oxidative stress [33–35]. Inspiration of high oxygen concentrations over time leads to increased oxidative stress brought about by an increase in the levels of reactive oxygen-derived free radicals, leading to generalized and localized inflammation, endothelial cell injury, and increased capillary permeability; this ultimately leads to acute lung injury, including VALI [36, 37]. Malondialdehyde is produced by lipid peroxidation of polyunsaturated fatty acids; MDA concentration helps estimate the degrees of lipid peroxidation and tissue injury [38–40]. In mammals, including humans, NO is present as a free radical; it is an important cellular signaling molecule, involved in many physiological and pathological processes [41]. Low NO production is important for protection of organs such as the lungs from ischemic damage [42]. Superoxide dismutase is the strongest oxygen radical-scavenging enzyme; it is widely distributed in the lungs, where it protects the lung tissue from injury. In theory, release of reactive oxygen species could enhance the expression of inflammatory mediators by increasing the concentrations of local and circulating cytokines [43, 44]. Our findings are consistent with those of previous studies in that, by the end of surgery, the plasma and BALF concentrations of MDA and NO had increased significantly, while the concentrations of SOD and inflammatory mediators had decreased in both groups. However, relative to the CV group, the PV group exhibited lower plasma and BALF concentrations of MDA and NO and higher BALF concentrations of SOD. On the basis of these results, we speculate that LPV with 6 mL/kg V_T_ and 10 cm H_2_O PEEP might result in lower systemic and local oxidative stress than conventional MV with 12 mL/kg V_T_ and 0 cm H_2_O PEEP.

Although the intergroup differences in inflammation and oxidative stress during surgery may not seem numerically impressive, clinical significance may present in patients undergoing craniotomy surgery. However, to prevent infection, the two hospital neurosurgeons practice different intraoperative and postoperative management methods compared to the standard international treatment protocol, especially in terms of antibiotic usage; this practice might hide some complications due to intraoperative inflammation and oxidative stress. For this reason, the PACU and Neurosurgery ICU stay times, cost, and prognosis were not observed in the present study. Nonetheless, other studies have reported that in patients with intermediate to high risk of pulmonary complications after a major abdominal surgery, intraoperative LPV with lower V_T_ (6–8 mL/kg PBW) with PEEP resulted in lower postoperative mortality than ventilation with higher V_T_ (10–12 mL/kg PBW) and no PEEP [14].

The traditional intraoperative two-lung ventilation technique of using V_T_ > 10 mL/kg PBW without PEEP is commonplace [45], although, in patients receiving one-lung ventilation for thoracic procedures, low V_T_ ventilation in conjunction with PEEP has been an accepted anesthetic practice since many years. There is now sufficient evidence to suggest the benefit of protective ventilation for short-term management of patients receiving general anesthesia. A multicenter study of more than 2900 patients receiving general anesthesia reported that 18% of patients were ventilated with V_T_ > 10 mL/kg PBW and 81% without PEEP [46]. Taken together, these findings suggest that, in the operating room, the concept of protective ventilation may rather be considered nonharmful during anesthesia in an otherwise healthy lung. Nonharmful ventilation might be an important concept for reducing pulmonary complications, and its design should, therefore, be an interesting field for further research [47].

There are several limitations to our study. Since this was a bicenter trial, surgery was performed by two different surgical teams, which would obviously have contributed to marginal procedural variability. To minimize this interinstitutional difference, we followed standardized anesthesiological management practices, while the surgical procedure was discussed, and decisions were made by consensus between the two surgical teams. Although the present sample size was adequate for achieving significant differences in endpoints between the two groups, it was not specifically powered to detect the effects of LPV in patients undergoing craniotomy. Finally, inclusion of another group of patients ventilated with low V_T_ and at high frequency without PEEP could have enriched this study. Thus, future studies involving larger sample sizes, other measurement indexes (e.g., intrapulmonary shunt rate), and an additional group of patients ventilated with low V_T_ and at high frequency without PEEP are required for evaluating the effects of LPV in individual patients.

## 5. Conclusions

In summary, our findings indicate that LPV with 6 mL/kg V_T_ and 10 cm H_2_O PEEP during craniotomy could cause relatively less VALI, without influencing the hemodynamic parameters, and also attenuate localized and generalized inflammatory responses and oxidative stress. Overall, LPV appears to be more desirable than conventional MV for minimizing the risk of VALI in the operating room in case of high-risk patients and/or prolonged anesthesia. Furthermore, LPV strategies need to be adjusted to suit individual patients.

## Figures and Tables

**Table 1:** Patient characteristics and intraoperative data.

Characteristics	Treatment groups		
	CV group (*n* = 27)	PV group (*n* = 28)	*P* value
Age, years	47 (10)	48 (10)	0.782
Sex, M/F	14/13	15/13	0.898
Weight, kg	68 (9)	66 (9)	0.346
Height, cm	167 (8)	168 (7)	0.765
ASA class I/II	10/17	9/19	0.703
Smoking habit	4 (15%)	5 (18%)	NS
Procedures			
Meningioma	12 (44%)	13 (46%)	NS
Glioma	7 (26%)	8 (29%)	NS
Intracranial aneurysm	8 (30%)	7 (25%)	NS
Duration of mechanical ventilation, min	400.8 (43.8)	408.3 (52.3)	0.549
Duration of surgery, min	347.2 (48.0)	348.5 (56.7)	0.928

Values are given as mean ± standard deviation or number of patients (%). CV group, conventional mechanical ventilation with 12 mL/kg tidal volume (V_T_) and 0 cm H_2_O positive end-expiratory pressure (PEEP); PV group, protective lung ventilation with 6 mL/kg V_T_ and 10 cm H_2_O PEEP. ASA class, American Society Anesthesiologists' physical status.

**Table 2:** Intraoperative blood loss, liquid input, and urine output.

Characteristics	Treatment groups		
	CV group (*n* = 27)	PV group (*n* = 28)	*P* value
Amount of bleeding, mL	424 (75)	420 (68)	0.868
Colloidal solution, mL	916 (271)	955 (277)	0.655
Crystalloid solution, mL	1635 (204)	1682 (198)	0.460
Transfusion of red blood cells, *n*	3 (11%)	4 (14%)	NS
Transfusion of plasma, *n*	2 (7%)	3 (11%)	NS
Urine output, mL	1741 (264)	1764 (308)	0.793

Values are given as mean ± standard deviation or number of patients (%). CV group, conventional mechanical ventilation with 12 mL/kg tidal volume (V_T_) and 0 cm H_2_O positive end-expiratory pressure (PEEP); PV group, protective lung ventilation with 6 mL/kg V_T_ and 10 cm H_2_O PEEP.
